# Comparative Outcomes of Single-Level Lumbar Laminectomy versus Hemilaminectomy: A Retrospective TriNetX Analysis

**DOI:** 10.1177/21925682261424530

**Published:** 2026-02-05

**Authors:** Christopher Sollenberger, Albert Q. Wu, Zachary Hoglund, Varun G. Kathawate, William Welch, Ali Ozturk, John Shin, Brendan F. Judy

**Affiliations:** 1 Department of Neurosurgery, Perelman School of Medicine, 6572 University of Pennsylvania, Philadelphia, PA, USA

**Keywords:** laminectomy, hemilaminectomy, radiculopathy, big data

## Abstract

**Study Design:**

Retrospective Cohort Study.

**Objectives:**

To compare 1-year postoperative outcomes and complication rates between single-level lumbar laminectomy and hemilaminectomy using a large, multicenter, propensity-matched dataset.

**Methods:**

We queried the TriNetX global health research network (≥160 million patients) for adults undergoing single-level lumbar decompression between January 2005 and July 2025. Cohorts were defined by CPT codes: laminectomy and hemilaminectomy, with qualifying diagnoses of lumbar disc herniation, spinal stenosis, spondylolisthesis, or radiculopathy. Patients with fusion, prior lumbar surgery, or non-degenerative pathology were excluded. Outcomes included new postoperative events within 1 year: mortality, weakness, pain, sensory loss, cauda equina syndrome, radiculopathy, foot drop, CSF leak, and surgical-site infection. Propensity-score matching balanced demographics and comorbidities. Cox proportional hazards models, Kaplan–Meier curves, and relative risks were calculated.

**Results:**

Of 167,177 patients, 80,440 underwent laminectomy and 86,737 hemilaminectomy. After matching, 50,853 patients per cohort were analyzed. One-year mortality was similar (0.57% vs 0.49%, HR 1.20; 95% CI 1.01-1.42; *P* = 0.045). Laminectomy conferred significantly higher risks of CSF leak (1.41% vs 1.00%; RR 1.41), surgical-site infection (1.45% vs 1.00%; RR 1.45), cauda equina syndrome (0.36% vs 0.22%; RR 1.62), and persistent weakness (4.12% vs 3.67%; RR 1.12). Persistent radiculopathy was modestly less frequent after laminectomy (10.5% vs 12.0%; RR 0.87). Other outcomes, including pain and foot drop, were comparable.

**Conclusions:**

Hemilaminectomy was associated with lower perioperative complication rates compared to laminectomy, while laminectomy provided a modest reduction in persistent radiculopathy. These findings highlight a tradeoff between safety and decompressive efficacy, emphasizing the importance of patient-specific surgical selection.

## Introduction

**Figure 1.**
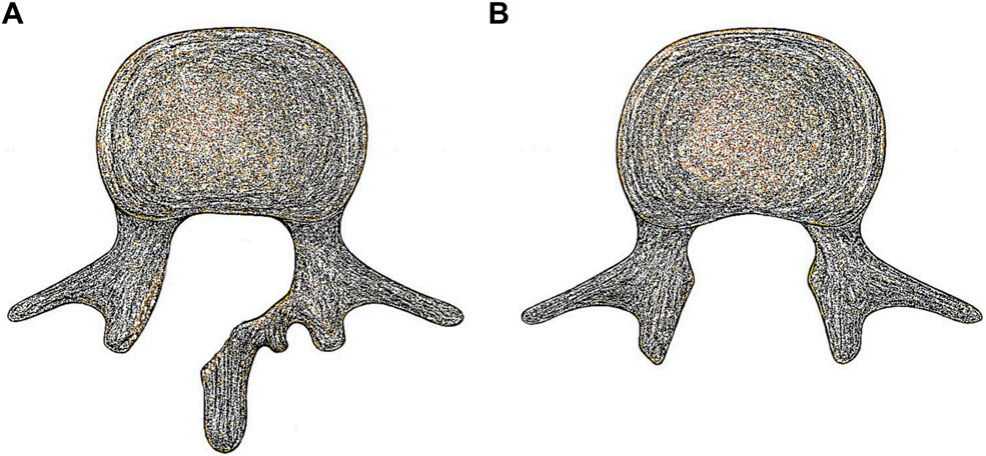
Illustrative example of (A) hemilaminectomy, which preserves the spinous process and contralateral lamina, and (B) laminectomy, which removes both laminae and the spinous process

Laminectomy and hemilaminectomy are well-established surgical procedures for decompressing the lumbar spine in conditions such as lumbar spinal stenosis and disc herniation (Figure 1).^1^ A laminectomy involves removal of the entire lamina and often the spinous process, providing a wide bilateral decompression of the neural elements.^2^ In contrast, a hemilaminectomy, or unilateral laminotomy, removes part of the lamina on one side and preserves the midline structures.^3^ Both techniques enlarge the spinal canal or neural foramen to relieve pressure on nerve roots, thereby alleviating radicular leg pain and neurogenic claudication in appropriately selected patients.^1^ These decompressive surgeries are standard options when conservative measures fail, and they share the same goal of achieving adequate nerve root decompression while minimizing damage to surrounding tissues with each approach offering distinct advantages and disadvantages. Additionally, these techniques both preserve the facet joints.

Laminectomy provides maximal exposure by removing the posterior bony arch, but this approach requires excision of the spinous process and interspinous ligaments which disrupts the posterior tension band, destabilizes the motion segment, and has been linked to greater blood loss, longer hospital stays, more postoperative pain, and a higher risk of instability requiring fusion or stabilization.^4,5^ Open laminectomy for lumbar stenosis has also been reported to result in significant soft tissue dissection and occasionally lead to segmental spinal instability necessitating instrumented fusion.^6,7^ Hemilaminectomy, on the other hand, is a tissue-sparing approach that removes only one lamina, preserving midline structures, and surrounding musculature.^3^ The preserved stability elements translate to a lower likelihood of postoperative spinal instability compared to a full laminectomy.^8^ Thus, hemilaminectomy has been credited with shorter operative times, less blood loss, and faster recoveries in some series due to the minimized muscle dissection.^9,10^ However, the narrower hemilaminectomy surgical corridor and limited exposure can make it technically more challenging to ensure complete decompression of neural elements which slightly raises the risk of inadvertent dural tears or nerve root injury.^11^ There is also a concern that an incomplete decompression with a limited approach could lead to residual or recurrent radiculopathy, necessitating revision surgery on the untreated side.^9^

Numerous prior studies have compared hemilaminectomy with traditional laminectomy for lumbar spinal stenosis, generally finding equivalent relief of leg and back pain but differences in complication profiles.^1,12^ Single-center reports also suggest lower incidental dural tear and infection rates with hemilaminectomy vs laminectomy, though these findings derive from older, small, single-center cohorts which may lack statistical power or adequately control for confounding variables.^9,13^ This underscores that while existing studies demonstrate comparative efficacy between laminectomy and hemilaminectomy, they have inherent limitations which highlights the need for a large, multicenter, propensity-matched trial to generate adequately powered, contemporary evidence on comparative outcomes.

The present study addresses this research gap by conducting a retrospective analysis using extensive data from the TriNetX database. TriNetX is a global network of real-world health care data encompassing over 160 million patients, which has been validated in recapturing outcomes from other established databases like the Nationwide Readmissions Database for various neurological and neurosurgical conditions.^14,15^ In this study, we specifically focused on examining patients who underwent single-level lumbar laminectomy or hemilaminectomy with 1-year follow-up to compare population-level outcome differences between the two procedures.

## Methods

### Data Source

We conducted a retrospective cohort study using the TriNetX platform (TriNetX, LLC, Cambridge, MA), a federated health research network that aggregates de-identified electronic health record data from dozens of healthcare organizations in the world.^16^ The platform extracts structured data including demographics, diagnoses, procedures, medications, and lab results, and applies natural language processing to capture relevant information from clinical notes. Prior to analysis, TriNetX harmonizes and standardizes data elements across sites, performs extensive preprocessing to address missing values, and enforces quality checks to ensure completeness and consistency of the research dataset.^17^

### Cohort Definition

**Figure 2.**
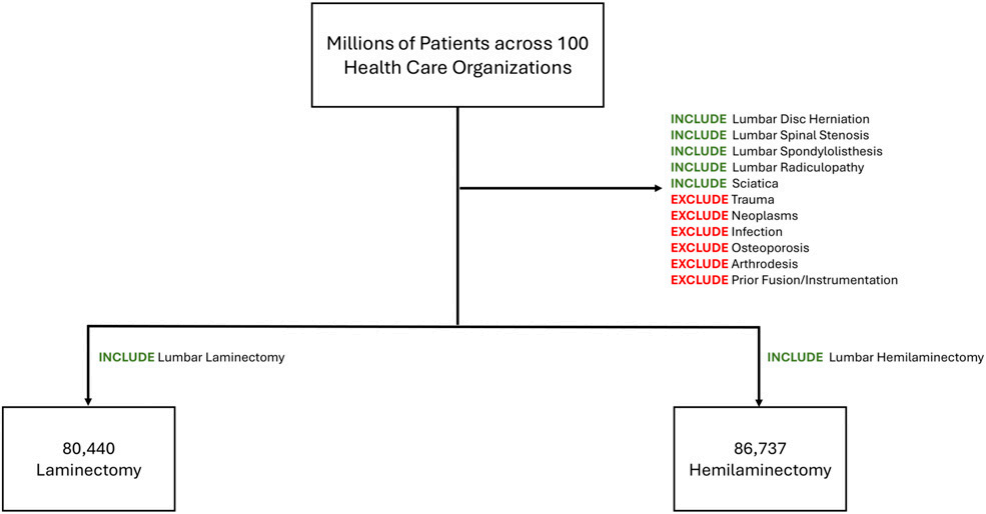
Flow chart of patient cohort assembly

We identified adults aged ≥18 years who underwent a single-level lumbar decompression between January 1, 2005 and July 31, 2025. Two cohorts were created based on CPT codes. The laminectomy cohort included patients with CPT code 63047, which codes for laminectomy with facetectomy/foraminotomy, and a qualifying diagnosis of lumbar disc herniation, spinal stenosis, spondylolisthesis, or radiculopathy. The hemilaminectomy, cohort was defined as patients with CPT code 63030, which identifies patients with hemilaminectomy/laminotomy/microdiscectomy, and the same diagnostic criteria. We excluded any patient with codes for lumbar fusion, nondegenerative spinal pathology—such as tumor, infection, acute fracture—or prior lumbar surgery. Index date was defined as the first qualifying procedure. Outcomes were tracked from postoperative day 1 through day 365. We measured the following new events: mortality, weakness, pain, sensory loss, cauda equina syndrome, radiculopathy, foot drop, CSF leak/dural tear, and surgical-site infection. To count an outcome, we required that the patient had no record of that condition before surgery. The ICD-10 and CPT codes used to define the cohorts and each outcome are shown in Figure 2 and listed in Appendix A: Cohort and Complications Definitions.

### Statistical Analysis

All statistical analyses were performed within the TriNetX platform. Propensity scores were generated using baseline demographics (age, sex, race), body mass index, tobacco use, and key comorbidities including hypertension, diabetes mellitus, metabolic disorders, ischemic heart disease, liver disease, neoplasms, opioid-related disorders, and alcohol-related disorders. Baseline characteristics were balanced using 1:1 propensity score matching via a greedy nearest‐neighbor algorithm with a caliper of 0.1 pooled standard deviations; balance was confirmed by standardized mean differences <0.1 for all covariates. For each presentation of an outcome we estimated hazard ratios (HR) and 95% confidence intervals (CI) using Cox proportional hazards models over the 1-year postoperative period, and generated Kaplan–Meier curves with log-rank tests to compare time-to-event distributions. Patients were censored at the time of last recorded follow-up or at 365 days postoperatively, ensuring appropriate handling of variable follow-up duration in all time-to-event analyses. We also computed complementary risk ratios and odds ratios for binary 1-year outcomes. All tests were two-sided with α = 0.05.

## Results

### Overall Cohort Description

A total of 167,177 patients met inclusion criteria. Of these, 80,440 underwent single-level lumbar laminectomy and 86,737 underwent hemilaminectomy. After 1:1 propensity-score matching on age, sex, race, body-mass index, tobacco use, and key comorbidities, two well-balanced cohorts of 50,853 patients each were analyzed (standardized mean differences <0.01 for all variables). A summary of patient characteristics before and after propensity matching is shown in Table 1. Mean follow-up was 292 ± 126 days in the laminectomy group and 304 ± 119 days in the hemilaminectomy group, with a median of 365 days for both.

**Table 1.** Demographic Characteristics of Patient Cohorts Before and After Propensity Matching

Variable	Before propensity matching		After propensity matching	
	Laminectomy (n = 80,440)	Hemilaminectomy (n = 86,737)	Laminectomy (n = 50,853)	Hemilaminectomy (n = 50,853)
Demographics				
Age (mean ± SD)	70.1 ± 13.6	57.4 ± 16.5	65.6 ± 14.0	65.7 ± 14.1
White (n%)	58,410 (74.3)	61,262 (74.0)	37,508 (73.8)	37,388 (73.5)
Black (n%)	5,090 (6.5)	4,369 (5.3)	3,121 (6.1)	3,174 (6.2)
Asian (n%)	2,104 (2.7)	1,959 (2.4)	1,270 (2.5)	1,223 (2.4)
Female (n%)	27,278 (34.7)	33,291 (40.2)	18,680 (36.7)	18,808 (37.0)
Baseline characteristics				
Overweight/Obesity (n%)	16,098 (20.5)	12,902 (15.6)	9,176 (18.0)	9,035 (17.8)
Hypertension (n%)	38,642 (49.1)	24,292 (29.3)	20,710 (40.7)	20,764 (40.8)
Diabetes (n%)	15,823 (20.1)	9,329 (11.3)	8,227 (16.2)	8,031 (15.8)
Chronic liver disease (n%)	5,164 (6.6)	3,860 (4.7)	3,005 (5.9)	2,922 (5.7)
Ischemic heart disease (n%)	13,217 (16.8)	6,358 (7.7)	6,205 (12.2)	5,971 (11.7)
Tobacco use (n%)	2,793 (3.6)	2,530 (3.1)	1,737 (3.4)	1,743 (3.4)
Opioid use disorder (n%)	1,221 (1.6)	1,094 (1.3)	764 (1.5)	703 (1.4)
Alcohol use disorder (n%)	2,389 (3.0)	2,137 (2.6)	1,496 (2.9)	1,430 (2.8)
Neoplasms (n%)	22,155 (28.2)	16,242 (19.6)	12,496 (24.6)	12,417 (24.4)
Chronic pain (n%)	25,686 (32.7)	20,680 (25.0)	14,536 (28.6)	14,226 (28.0)

### Mortality and Survival

Within one year, there were 288 deaths (0.57%) after laminectomy vs 250 deaths (0.49%) after hemilaminectomy (absolute difference .1%), indicating no statistically significant difference in 1-year mortality. Kaplan–Meier survival curves diverged slightly by 12 months (99.31% vs 99.41%; log-rank χ^2^ = 4.248, df = 1, *P* = 0.039), and the adjusted hazard ratio for death was 1.195 (95% CI 1.01-1.42; *P* = 0.045).

### Operative Complications

**Figure 3.**
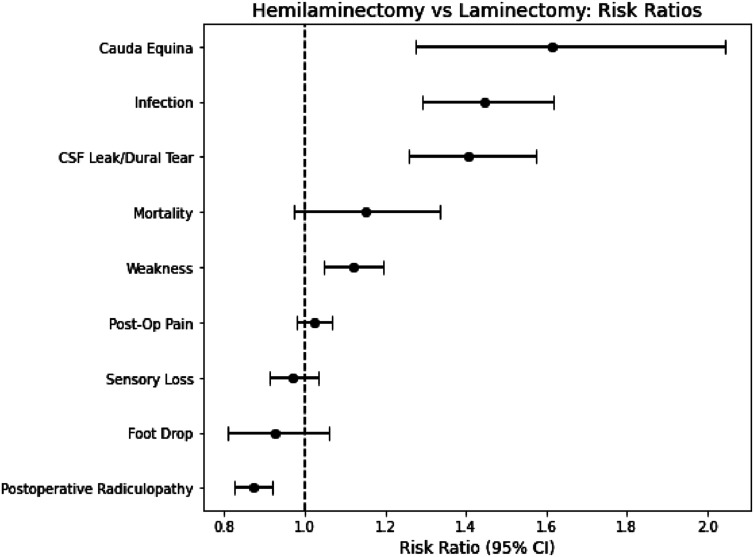
Risk ratios of 1-year postoperative complication rates between laminectomy and hemilaminectomy

In our propensity-matched analysis, hemilaminectomy was associated with significantly lower complication rates (Table 2) and favorable relative risks (Figure 3). Patients undergoing laminectomy experienced a 41% higher risk of CSF leak compared with hemilaminectomy (RR 1.41), corresponding to 719 vs 511 events (1.41% vs 1.00%; RD 0.4%, 95% CI 0.3%-0.5%; *P* < 0.001). Similarly, laminectomy carried a 45% greater relative risk of surgical-site infection (RR 1.45), with 725 infections (1.45%) vs 502 (1.00%) and an absolute increase of 0.4% (95% CI 0.3%-0.6%; *P* < 0.001). The relative risk of new cauda equina syndrome was 1.62 after laminectomy (179 events, 0.36% vs 112, 0.22%; RD 0.1%, 95% CI 0.1%-0.2%; *P* < 0.001). By contrast, foot drop rates were nearly identical (RR 0.926; 402 vs 434 patients; 0.83% vs 0.89%; RD −0.1%, 95% CI −0.2%-0.0%; *P* = 0.264). Laminectomy was also associated with a 12% higher relative risk of persistent weakness (RR 1.12), with 1788 patients (4.12%) affected in the laminectomy cohort vs 1610 in the hemilaminectomy cohort (3.67%; RD 0.4%, 95% CI 0.2%–0.7%; *P* = 0.001). Sensory loss was 3% less likely following laminectomy (RR 0.97) despite a small absolute increase after hemilaminectomy (4.57% vs 4.71%; RD –0.1%, 95% CI −0.4% to 0.1%; *P* = 0.331).

**Table 2.** Comparison of One-Year Postoperative Complication Rates Between Laminectomy and Hemilaminectomy

Variable	Laminectomy	Hemilaminectomy	Risk difference	95% CI	*P*-value
New complications (%)					
Mortality	0.6	0.5	0.001	0.000 - 0.001	0.1
Cauda Equina	0.4	0.2	0.001	0.001 - 0.002	<0.001
CSF Leak/Dural tear	1.4	1.0	0.004	0.003 - 0.005	<0.001
Infection	1.5	1.0	0.004	0.003 - 0.006	<0.001
Weakness	4.1	3.7	0.004	0.002 - 0.007	0.001
Sensory loss	4.6	4.7	−0.001	−0.004 - 0.001	0.331
Foot drop	0.8	0.9	−0.001	−0.002 - 0.000	0.264
Persistent complications (%)					
Pain	16.7	16.4	0.004	−0.003 - 0.011	0.293
Radiculopathy	10.5	12.0	−0.015	−0.022 - −0.009	<0.001

### Persistent Complications

Hemilaminectomy also conferred advantages for several persistent complications (Table 2; Figure 3). Persistent radiculopathy was slightly less frequent following laminectomy (10.49%) than hemilaminectomy (12.03%), RR 0.872 (95% CI 0.83-0.92) despite a small absolute reduction with laminectomy (RD −1.5%; 95% CI −2.2% to −0.9%; *P* < 0.001). Finally, new or worsening postoperative pain affected 3491 laminectomy patients (16.74%) and 3445 hemilaminectomy patients (16.36%), yielding an RR of 1.02 (95% CI 0.98-1.07) and a non-significant RD of −0.4% (95% CI −0.3% to 1.1%; *P* = 0.293).

## Discussion

Hemilaminectomy demonstrated a consistent safety advantage over traditional laminectomy across a broad range of perioperative complications, while generally maintaining decompressive efficacy. While these modest absolute differences may meaningfully influence outcomes when considered in aggregate across large populations, they are unlikely to manifest as readily observable differences at the level of individual providers or single institutions. Patients undergoing laminectomy were 41% more likely to sustain a dural tear (RR 1.41; 95% CI 1.26-1.58) and 45% more likely to develop a surgical-site infection (RR 1.45; 95% CI 1.29-1.62), each with an absolute increase of 0.4% (*P* < 0.001 for both; Table 2, Figure 3). The relative risk of cauda equina syndrome was 1.62 (95% CI 1.28-2.05; RD 0.1%; *P* < 0.001), indicating an increased risk of cauda equina syndrome following laminectomy. In contrast, rates of foot drop (RR 0.93) and postoperative pain (RR 1.02) did not differ meaningfully. Persistent weakness remained more common after laminectomy (RR 1.12; 95% CI 1.05-1.20; RD 0.4%; *P* = 0.001), whereas sensory deficits were slightly less likely (RR 0.97; 95% CI 0.91-1.03; RD –0.1%; *P* = 0.331). The only outcome modestly favoring laminectomy was radiculopathy, which was marginally lower (RR 0.87; 95% CI 0.83-0.92; RD −0.8%; *P* < 0.001), suggesting that the more extensive decompression may better resolve nerve-root compression.

These findings underscore that hemilaminectomy offers a lower complication risk profile but may be modestly less effective at fully addressing the primary indication of nerve decompression than full laminectomy. In practical terms, these findings support hemilaminectomy as a reasonable first-line decompression strategy for patients with focal or unilateral pathology and elevated risk for perioperative complications, while reserving full laminectomy for patients with more extensive, bilateral, or multilevel stenosis in whom maximal decompression is the primary goal. As such, patient selection is crucial: those with severe or multilevel stenosis may derive greater symptomatic relief from laminectomy despite its higher perioperative risks, whereas patients at elevated risk for dural tears, infection, or iatrogenic instability may be better served by a hemilaminectomy. Ultimately, surgeons must balance this tradeoff between safety and efficacy, tailoring the decompression technique to each patient’s anatomic pathology, comorbidity burden, and functional goals.

Moreover, several limitations warrant consideration. As a retrospective EHR-based study, our analysis depends on the accuracy and completeness of coding. Reliance on administrative database coding limits our ability to capture surgical nuance, including laterality, extent of decompression, and true clinical severity, which should be considered when interpreting the conclusions. Specifically, bundled CPT codes do not reliably distinguish microdiscectomy alone from hemilaminectomy or laminectomy with concomitant discectomy, precluding further stratification by operative subtype. Despite restriction to overlapping degenerative indications and propensity score matching, residual indication bias may persist, as hemilaminectomy and laminectomy may still be preferentially selected based on symptom laterality, anatomic severity, or surgeon judgment that cannot be fully captured in administrative data. Subtle clinical nuances, such as the exact extent of neural decompression or surgeon decision-making, are not captured. The 1-year follow-up, while sufficient to assess most early complications, may miss late reoperations for instability or recurrent stenosis. We also lacked standardized patient-reported outcomes to gauge functional recovery and quality of life, including direct measures of symptom resolution and patient satisfaction, which limits assessment of decompression efficacy. Unmeasured confounders could also influence both the choice of procedure and outcomes. Moreover, surgical selection bias may exist, as hemilaminectomies are typically performed for unilateral radiculopathy while bilateral laminectomies are reserved for stenosis, claudication, or bilateral radiculopathies; these underlying differences may influence outcomes, though our findings suggest unilateral hemilaminectomy may carry lower complication risks when feasible. Finally, although our large sample size allows us to detect statistically significant differences between hemilaminectomy and laminectomy, those absolute risk differences are small in practical terms—translating into only a few additional events per thousand patients rather than dramatic changes at the individual level.

Despite these constraints, the large, propensity-matched sample across multiple centers strengthens the generalizability of our findings. Future prospective research should integrate radiographic data, long-term follow-up, and validated patient-reported measures to better define which patients gain the greatest net benefit from hemilaminectomy vs full laminectomy, and to inform effectiveness in spine care.

## Conclusion

In summary, our large retrospective analysis indicates that hemilaminectomy substantially lowers the risk of operative complications, but it may be modestly less effective at fully resolving patients’ symptoms. This tradeoff between safety and efficacy underscores the importance of tailoring the surgical approach to each patient’s anatomic severity, comorbidities, and functional goals. Careful patient selection is essential to balance the lower complication rates of hemilaminectomy against its slightly higher likelihood of persistent radiculopathy. Future prospective studies incorporating imaging severity, long-term follow-up, and patient-reported outcomes will be critical to refine decompression strategies and optimize both safety and effectiveness.

## Supplemental Material

Supplemental Material - Comparative Outcomes of Single-Level Lumbar Laminectomy versus Hemilaminectomy: A Retrospective TriNetX Analysis Supplemental Material for Comparative Outcomes of Single-Level Lumbar Laminectomy versus Hemilaminectomy: A Retrospective TriNetX Analysis by Christopher Sollenberger, Albert Q. Wu, Zachary Hoglund, Varun G. Kathawate, William Welch, Ali Ozturk, John Shin, Brendan F. Judy in Global Spine Journal.

## Data Availability

No new data were generated or analyzed in this study.*

## References

[1] Williams MG Wafai AM Podmore MD . Functional outcomes of laminectomy and laminotomy for the surgical management lumbar spine stenosis. J Spine Surg. 2017;3(4):580-586. doi:10.21037/jss.2017.10.08 29354735 PMC5760430

[2] Ross N Aleman C Dhenin A Vassal M Lonjon G . Tubular versus unilateral biportal endoscopy: MRI analysis after unilateral laminectomy for bilateral decompression in lumbar spinal stenosis. Eur Spine J. 2025;34:2972-2980. doi:10.1007/s00586-025-08953-3 40448852

[3] Sun CX Meng XL Xie SN Yu Y Yang HJ Wu B . Unilateral hemilaminectomy for patients with intradural extramedullary tumors. J Zhejiang Univ - Sci B. 2011;12(7):575-581. doi:10.1631/jzus.B1000402 21726065 PMC3134846

[4] Lee MJ Bransford RJ Bellabarba C , et al. The effect of bilateral laminotomy versus laminectomy on the motion and stiffness of the human lumbar spine: a biomechanical comparison. Spine. 2010;35(19):1789-1793. doi:10.1097/BRS.0b013e3181c9b8d6 20562732

[5] Lai PL Chen LH Niu CC Fu TS Chen WJ . Relation between laminectomy and development of adjacent segment instability after lumbar fusion with pedicle fixation. Spine. 2004;29(22):2527-2532. doi:10.1097/01.brs.0000144408.02918.20. discussion 2532. 15543067

[6] Guha D Heary RF Shamji MF . Iatrogenic spondylolisthesis following laminectomy for degenerative lumbar stenosis: systematic review and current concepts. Neurosurg Focus. 2015;39(4):E9. doi:10.3171/2015.7.Focus15259 26424349

[7] Schöller K Alimi M Cong GT Christos P Härtl R . Lumbar spinal stenosis associated with degenerative lumbar spondylolisthesis: a systematic review and meta-analysis of secondary fusion rates following open vs minimally invasive decompression. Neurosurgery. 2017;80(3):355-367. doi:10.1093/neuros/nyw091 28362963

[8] Mobbs RJ Maharaj MM Phan K Rao PJ . Unilateral hemilaminectomy for intradural lesions. Orthop Surg. 2015;7(3):244-249. doi:10.1111/os.12184 26311099 PMC6583753

[9] Phan K Mobbs RJ . Minimally invasive versus open laminectomy for lumbar stenosis: a systematic review and meta-analysis. Spine. 2016;41(2):E91-e100. doi:10.1097/brs.0000000000001161 26555839

[10] Sharma E da Silva Lobo KE Ayesha A , et al. Minimally invasive decompression versus open laminectomy in multilevel lumbar stenosis: a systematic review and meta-analysis. World Neurosurg. 2025;198:124031. doi:10.1016/j.wneu.2025.124031 40339745

[11] Dobran M Paracino R Nasi D , et al. Laminectomy versus unilateral hemilaminectomy for the removal of intraspinal schwannoma: experience of a single institution and review of literature. J Neurol Surg Cent Eur Neurosurg. 2021;82(6):552-555. doi:10.1055/s-0041-1722968 33845505

[12] Munting E Röder C Sobottke R Dietrich D Aghayev E Spine Tango Contributors . On behalf of the Spine Tango C. Patient outcomes after laminotomy, hemilaminectomy, laminectomy and laminectomy with instrumented fusion for spinal canal stenosis: a propensity score-based study from the Spine Tango registry. Eur Spine J. 2015;24(2):358-368. doi:10.1007/s00586-014-3349-0 24840246

[13] Overdevest GM Jacobs W Vleggeert-Lankamp C Thomé C Gunzburg R Peul W . Effectiveness of posterior decompression techniques compared with conventional laminectomy for lumbar stenosis. Cochrane Database Syst Rev. 2015;2015(3):Cd010036. doi:10.1002/14651858.CD010036.pub2 25760812 PMC11627146

[14] Wu A Davis P Scott K Catapano J Burkhardt J-K Srinivasan V . Reader Response: Efficacy and Safety of IV Thrombolysis for Acute Ischemic Stroke Patients with Moyamoya Disease; 2025.

[15] Chen H Colasurdo M Khunte M Malhotra A Gandhi D . Efficacy and safety of IV thrombolysis for acute ischemic stroke patients with Moyamoya disease. Neurology. 2025;104(3):e210243. doi:10.1212/wnl.0000000000210243 39772663

[16] Ludwig RJ Anson M Zirpel H , et al. A comprehensive review of methodologies and application to use the real-world data and analytics platform TriNetX. Front Pharmacol. 2025;16:1516126. doi:10.3389/fphar.2025.1516126 40129946 PMC11931024

[17] Palchuk MB London JW Perez-Rey D , et al. A global federated real-world data and analytics platform for research. JAMIA Open. 2023;6(2):ooad035. doi:10.1093/jamiaopen/ooad035 37193038 PMC10182857

