# Supplementary material for: Comparative Outcomes of Single-Level Lumbar Laminectomy versus Hemilaminectomy: A Retrospective TriNetX Analysis
Source: Global Spine J. 2026 Feb 5:21925682261424530. Online ahead of print. doi: 10.1177/21925682261424530 (PMC12875885; doi:10.1177/21925682261424530)
Supplement: Supplemental Material - Comparative Outcomes of Single-Level Lumbar Laminectomy versus Hemilaminectomy: A Retrospective TriNetX Analysis [file sj-pdf-1-gsj-10.1177_21925682261424530.pdf]

Appendix A. Cohort and Complications Definitions

1. Cohort Definitions

1.1 Common Eligibility (both cohorts)

Inclusion: Degenerative Lumbar Diagnoses
Patients must have **≥1** of these ICD-10-CM codes:

Code	Description
UMLS:ICD10CM:M51.26	Other intervertebral disc displacement, lumbar region
UMLS:ICD10CM:M51.27	Other intervertebral disc displacement, lumbosacral
UMLS:ICD10CM:M48.06	Spinal stenosis, lumbar region
UMLS:ICD10CM:M48.07	Spinal stenosis, lumbosacral region
UMLS:ICD10CM:M43.16	Spondylolisthesis, lumbar region
UMLS:ICD10CM:M43.17	Spondylolisthesis, lumbosacral region
UMLS:ICD10CM:M54.16	Radiculopathy, lumbar region
UMLS:ICD10CM:M54.17	Radiculopathy, lumbosacral region
UMLS:ICD10CM:M54.30	Sciatica, unspecified side
UMLS:ICD10CM:M54.31	Sciatica, right side
UMLS:ICD10CM:M54.32	Sciatica, left side

Exclusion: Non-degenerative or Fusion Procedures
Exclude if any of these codes appear:

Code Type	Code(s)	Description
ICD-10-CM	UMLS:ICD10CM:S32*	Fracture of lumbar spine/pelvis
ICD-10-CM	UMLS:ICD10CM:S33*	Dislocation/sprain, lumbar spine
ICD-10-CM	UMLS:ICD10CM:S34.3, UMLS:ICD10CM:S34.4	Cauda equina/lumbosacral plexus injury
ICD-10-CM	UMLS:ICD10CM:C41.2, UMLS:ICD10CM:C79.51, UMLS:ICD10CM:D16.6	Spinal neoplasms
ICD-10-CM	UMLS:ICD10CM:M46.46, UMLS:ICD10CM:M46.47	Discitis
ICD-10-CM	UMLS:ICD10CM:G06.1	Intraspinal abscess
ICD-10-CM	UMLS:ICD10CM:A40.3	Streptococcus pneumoniae sepsis
ICD-10-CM	UMLS:ICD10CM:M80.08XA, UMLS:ICD10CM:M81.8, UMLS:ICD10CM:Z87.310	Osteoporosis fractures/history

Code Type	Code(s)	Description
CPT / PCS	UMLS:CPT:22633, UMLS:CPT:22612, UMLS:CPT:22840–22842	Arthrodesis/instrumentation (fusion)
ICD-10-PCS	UMLS:ICD10PCS:0SG30AJ, UMLS:ICD10PCS:0SG33J1	Lumbosacral fusion

1.2 Procedure-Based Cohorts

Cohort	CPT Code	Description
Lumbar Laminectomy	UMLS:CPT:63047	Laminectomy + facetectomy + foraminotomy, 1 segment, lumbar
Hemilaminectomy (Laminotomy)	UMLS:CPT:63030	Unilateral laminotomy/hemilaminectomy + partial facetectomy/foraminotomy, 1 segment

Index date = first recorded procedure code.

---

2. Complication (Outcome) Definitions

Each outcome is defined by ≥1 diagnosis or procedure code occurring 1–365 days post-index, excluding patients with prior history of that code.

CSF Leak / Dural Tear

Code Type	UMLS Code	Description
Procedure	UMLS:CPT:63707	Repair CSF leak (no laminectomy)
Procedure	UMLS:CPT:63709	Repair CSF leak/pseudomeningocele (with laminectomy)
Diagnosis	UMLS:ICD10CM:G96.0	Cerebrospinal fluid leak
Diagnosis	UMLS:ICD10CM:G97.41	Accidental puncture/laceration of dura
Diagnosis	UMLS:ICD10CM:G97.49	Accidental puncture/laceration of nervous structure

Surgical-Site Infection

Code Type	UMLS Code	Description
Procedure	UMLS:CPT:10180	Incision & drainage, complex, postoperative infection
Procedur	UMLS:CPT:22010	Incision & drainage, deep abscess, posterior

Code Type	UMLS Code	Description
e		spine
Procedure	UMLS:CPT:10060, 10140	I&D of abscess or fluid collection
Diagnosis	UMLS:ICD10CM:M86.08	Acute hematogenous osteomyelitis
Diagnosis	UMLS:ICD10CM:M46.46	Discitis, lumbar
Diagnosis	UMLS:ICD10CM:G06.1	Intraspinal abscess
Diagnosis	UMLS:ICD10CM:T81.4XXA	Infection following procedure

Cauda Equina Syndrome

Code Type	UMLS Code	Description
Diagnosis	UMLS:ICD10CM:G83.4	Cauda equina syndrome

Foot Drop / Motor Defects

Code Type	UMLS Code	Description
Diagnosis	UMLS:ICD10CM:M21.371	Foot drop, right foot
Diagnosis	UMLS:ICD10CM:M21.372	Foot drop, left foot
Diagnosis	UMLS:ICD10CM:M21.379	Foot drop, unspecified

Persistent Weakness

Code Type	UMLS Code	Description
Diagnosis	UMLS:ICD10CM:G81.90	Hemiplegia, unspecified
Diagnosis	UMLS:ICD10CM:M62.81	Muscle weakness (generalized)
Diagnosis	UMLS:ICD10CM:R53.1	Weakness
Diagnosis	UMLS:ICD10CM:R26.2	Difficulty walking

Sensory Loss

Code Type	UMLS Code	Description
Diagnosis	UMLS:ICD10CM:R20.*	Skin sensation disturbances
Diagnosis	UMLS:ICD10CM:R20.1	Hypoesthesia of skin
Diagnosis	UMLS:ICD10CM:R20.2	Paresthesia of skin

Code Type	UMLS Code	Description
Diagnosis	UMLS:ICD10CM:G58.9	Mononeuropathy, unspecified

Postoperative Radiculopathy

Code Type	UMLS Code	Description
Diagnosis	UMLS:ICD10CM:M54.16	Radiculopathy, lumbar region
Diagnosis	UMLS:ICD10CM:M54.17	Radiculopathy, lumbosacral region
Diagnosis	UMLS:ICD10CM:G55	Nerve root/plexus compression

Post-Op Pain

Code Type	UMLS Code	Description
Diagnosis	UMLS:ICD10CM:M96.1	Postlaminectomy syndrome
Diagnosis	UMLS:ICD10CM:G89.4	Chronic pain syndrome
Diagnosis	UMLS:ICD10CM:G89.29	Other chronic pain
Diagnosis	UMLS:ICD10CM:M54.5	Low back pain
Diagnosis	UMLS:ICD10CM:R52	Pain, unspecified

Mortality

Code Type	Description (Demographics)
Deceased	Deceased status recorded in EHR
